# Supplementary material for: Loss of Proximal Tubular Sirtuin 6 Aggravates Unilateral Ureteral Obstruction-Induced Tubulointerstitial Inflammation and Fibrosis by Regulation of β-Catenin Acetylation
Source: Cells. 2022 Apr 27;11(9):1477. doi: 10.3390/cells11091477 (PMC9100256; doi:10.3390/cells11091477)
Supplement: Supplementary file 1 [file cells-11-01477-s001.zip › cells-1602822-supplementary.pdf]

Supplementary data

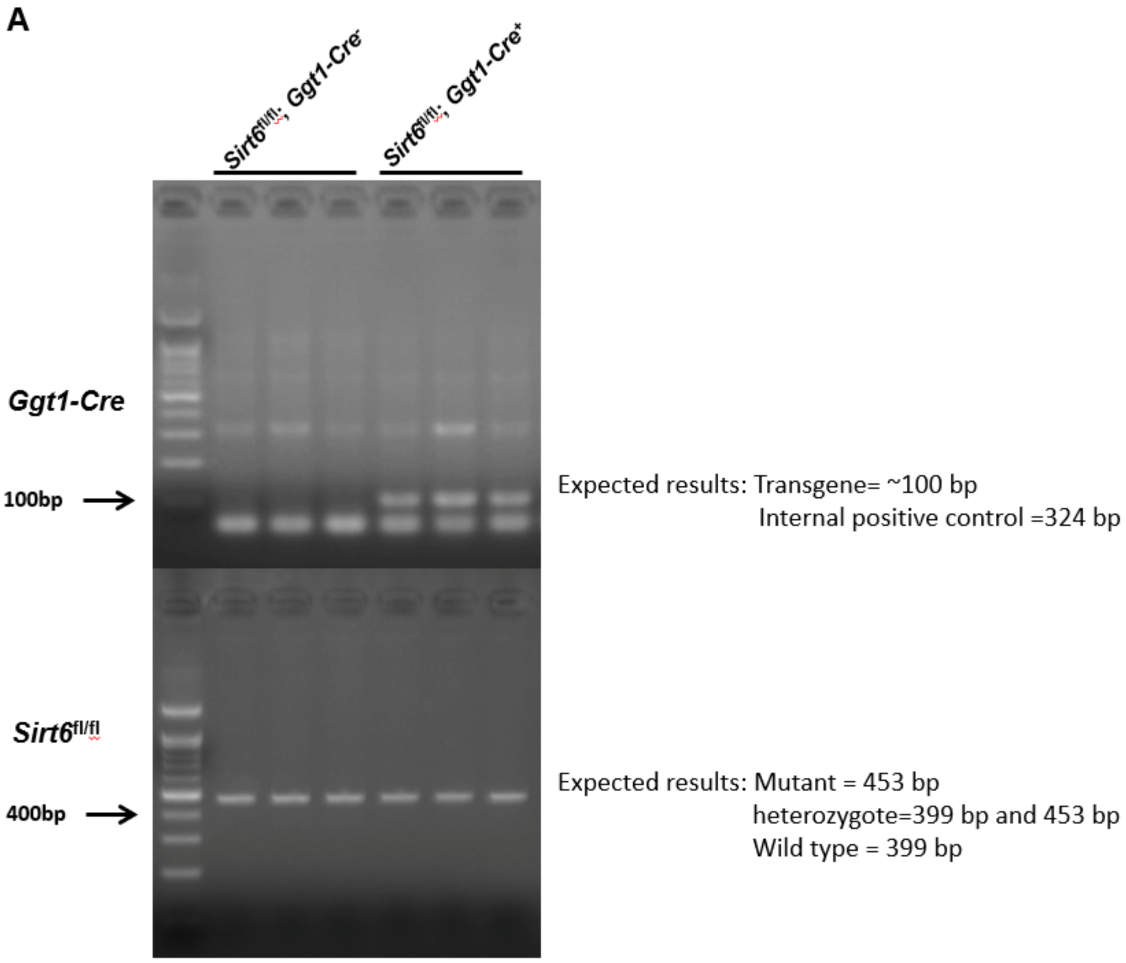

Figure S1. Genotype for *PT*-sirt6KO mice. Representative RT-PCR for proximal tubule-specific Sirt6 knockout mice after crossing *Sirt6*$^{flox/flox}$ (*Sirt6*$^{tm1.1Cxd}$/J) and homozygous γ-glutamyl transferase Cre mice (*Tg-(Ggt1-Cre)M3Egn/J*).
